# Modeling of a Novel Approach to Water Recovery from Sugar Cane Vinasse: Optimization of Exergy Efficiency, Heat Exchange Area, and Thermal Performance Ratio Using Genetic Algorithm and TOPSIS

**DOI:** 10.1021/acsomega.5c04917

**Published:** 2025-09-14

**Authors:** Thúlio B. Oliveira, Esly F. Costa Junior, Andréa O. S. Costa

**Affiliations:** Escola de Engenharia, Universidade Federal de Minas Gerais—UFMG, Belo Horizonte 31270-901, Brazil

## Abstract

Historically, multistage flash distillation (MSF) led potable water production. By 2019, emerging technologies largely replaced MSF; however, it remains attractive for its energy and exergetic efficiency due to its reliance on low-temperature heat sources, especially when cogeneration is involved. In contrast, the sugar-energy industry consumes around 0.7 m^3^ of water per metric ton of sugar cane processed. Rising environmental, social, and corporate governance (ESG) concerns have intensified efforts to reduce water use. Accordingly, this study identified an optimal configuration for integrating a once-through MSF (MSF-OT) system with a sugar cane processing facility. The proposed system recovers water from vinasse, a byproduct of ethanol production, by using steam generated during the sugar cane juice concentration process as its energy source. The process was modeled in MATLAB using data from a Brazilian plant, and optimization was performed with a genetic algorithm targeting exergy efficiency, total heat exchange area, and thermal performance ratio. The Order Preference by Similarity to Ideal Solution (TOPSIS) method subsequently selected the best alternative from the Pareto Front. Results indicated that 58 flashing stages and a terminal temperature drop in the condenser of the first stage of 4.3 °C are the optimal configuration, producing 50,080 m^3^ of distilled water per harvest.

## Graphical abstract

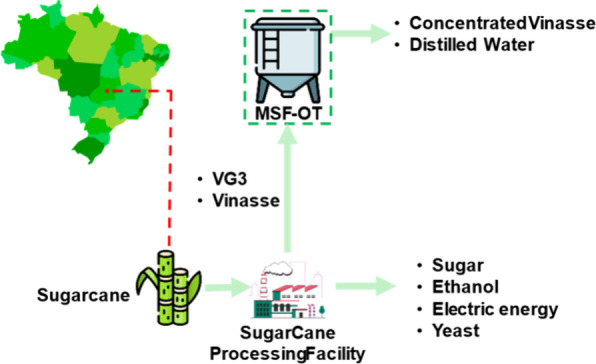

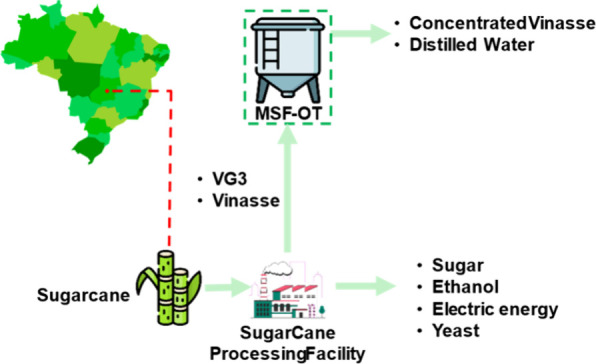

## Introduction

Multistage flash distillation (MSF) has historically been the most widely employed technique for seawater desalination worldwide, accounting for over 60% of all desalination operations at the beginning of the 21st century (El-Dessouky and Ettouney^1^). However, its prevalence has declined considerably in recent years; by 2019, it constituted only 18% of desalination operations, having been largely supplanted by reverse osmosis desalination (Jones et al., 2019 ^2^).

MSF remains viable when the fuel used is sufficiently inexpensive (Al-Mutaz^3^). In the Gulf region, this technology is currently predominant, primarily due to the abundant availability of fossil fuels and their relatively low extraction costs (Aleisa^4^). Furthermore, because this external thermal source operates at relatively low temperatures (generally below 100 °C), the MSF process is an excellent candidate for integration with cogeneration systems (Kahraman and Cengel^5^).

MSF is also applied for treating industrial wastewater. Tayyeban et al.^6^ proposed two models and multiobjective optimization routines for a once-through multistage flash (MSF-OT) desalination plant and an MSF plant with thermal vapor compression (MSF-TVC), with the objective of treating an industrial effluent from the Hashemi Nejad refinery, located in the city of Sarakhs in the Iranian province of Khorasan Razavi.

Consequently, MSF is not only viable but can also be the optimal choice when it can be operated in conjunction with another industrial process that provides either cogeneration capacity or low-temperature steam availability. Such integration can enhance the overall energy efficiency of the integrated process and reduce the environmental impacts associated with water resource pollution. Moreover, if the distilled water is recirculated within the process, the required water consumption is reduced, together with the energy needed for water pumping.

The sugar-energy sector spans the entire production cycle of sugar cane, which serves as the raw material for different grades of sugar, yeast, ethanol, and electrical energy derived from the combustion of sugar cane bagasse. Alongside these products, the process also yields vinasse, a byproduct that presents environmental challenges but offers promising opportunities for water recovery. Defined as the residue at the bottom of the distillation column used in ethanol production, vinasse exhibits high levels of organic matter and nutrients (Silveira^7^) and is approximately 93% water (Spinelli et al.^8^). Its rich organic matter, nutrient load, and high-water content is particularly well-suited for biogas production through biodigesters and for use in fertigation systems. Another potential application of vinasse lies in concentration systems designed to minimize its volume, thereby improving transport logistics and allowing for the recovery of water.

Cruz et al.^9^ evaluated these three vinasse applications from a technical, environmental, and economic perspective. The concentration process proved to be environmentally viable, thanks to the water reuse it enables and the reduced environmental impact due to the lower volume of vinasse. It was also found to be technically feasible. However, the authors concluded that, from an economic standpoint, the vinasse concentration technologies currently available are not viable.

Nevertheless, several plants have vinasse concentration methods in their operations. The majority rely on a multiple-effect evaporation process, which is driven by high temperature steam (Cruz et al.^9^). Despite the substantial volume of low-temperature steam generated during the concentration of sugar cane juice by the multistage Robert-type evaporation system, commonly used in Brazilian sugar-energy plants, studies examining the feasibility of implementing an MSF system in conjunction with a sugar-energy plant remain scarce at the time of writing. Therefore, this work investigated the feasibility of integrating an MSF-OT system for vinasse concentration using VG3 (a low temperature steam from the concentration of sugar cane juice in saturation state) as the heat source.

## Methodology

### Model Assumptions

The model was developed based on the mass and energy balance of the proposed system for vinasse concentration using VG3, employing the commercial software MATLAB. This model aims to simulate the operating conditions of the proposed plant based on the selected input data and constraints. Correlations and other equations intrinsic to the modeling of MSF-OT processes were also utilized.

The data used in this work were obtained from a sugar cane processing facility in Goianésia, Brazil. The obtained values represent the average of these parameters during the plant operation. The data is presented below:

- *T*_S_ (VG3 temperature): 98 °C
- *ṁ*_F_ (Feed Stream mass flow rate): 62 kg/s
- *X*_F_ (Feed Stream solids concentration): 38,136 ppm
- *T*_CW_ (Feed Stream initial temperature): 61 °C

The input variables and their respective sources are listed below (Table 1):

**Table 1:** Input Variables and Their Respective Sources

parameter	input origin
*T*_*S*_	industrial data
*ṁ*_*F*_	industrial data
*X*_F_	industrial data
*T*_*CW*_	industrial data
*n*	variable to be optimized
TTD_*c*_(1)__	variable to be optimized
TTD_*h*_	literature
Δ*T*_non_	literature
Δ*T*_demister_	literature
η_*TC*_	literature

The following assumptions are adopted for the mass and energy balance of the proposed system. First, it is considered to operate under steady-state conditions. For simplification, vinasse is assumed to be a binary solution composed solely of sucrose and water. The presence of noncondensable gases is neglected, and the steam generated during the process is considered to consist only of water.

The following assumptions are adopted for the mass and energy balance of the proposed system. It is considered to operate under steady-state conditions. For simplification, vinasse is assumed to be a binary solution composed solely of sucrose and water. The steam generated during the process is considered to consist only of water. The presence of noncondensable gases is neglected, although it is important to note that these gases can significantly impair the heat exchange process by hindering steam condensation. If not effectively removed, they may lead to increased stage pressure, ultimately disrupting the flashing process.

Within each flashing stage, the concentration is assumed to be uniform, with no occurrence of chemical reactions throughout the process. Additionally, it is assumed that there are no mass losses within the system. Pressure and temperature remain constant and uniform in every flashing stage, while variations in kinetic and potential energy are considered negligible.

It is also assumed that all steam generated in each flashing stage is entirely condensed. Furthermore, the steam utilized for heating purposes fully condenses and does not exchange sensible heat with the incoming vinasse stream. The sensible heat transferred by the condensed water entering a flashing stage is deemed negligible in the computation of the total heat exchange area of the condensers.

The overall heat transfer coefficient is taken to be uniform across all heat exchangers. Moreover, the work required to operate both the vacuum-producing exhauster and the system’s feed pump is disregarded for simplification. Finally, the efficiency of the heat exchangers (η_TC_) is assumed to be 90%, based on the methodology presented by Amiri Rad and Mohammadi.^10^

### Mass and Energy Balances

The proposed system is shown in Figure 1, with the control volumes (C.V.) that are used to model the process:

**Figure 1:**
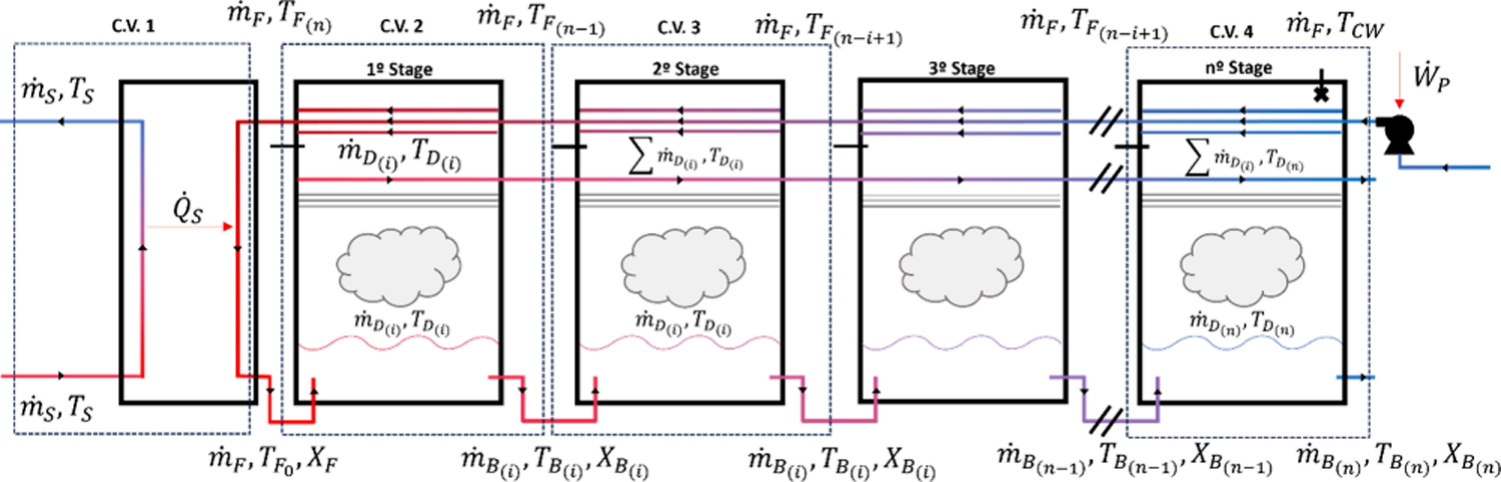
Process diagram with the used C.V. for the modeling of the proposed system.

The following eqs 1–5 describe the mass balance in the proposed system:

For the steam flow responsible for heating the system (C.V.1):

$${\dot{m}}_{S_{\mathrm{in}}}={\dot{m}}_{S_{\mathrm{out}}}$$ 1

Note that the symbols in and out were used to represent, respectively, the steam inlet flow and the condensate outlet flow

For any flashing stage (C.V.3):

$${ṁ}_{B_{\left(i-1\right)}}={ṁ}_{B_{\left(i\right)}}+{ṁ}_{D_{\left(i\right)}}$$ 2

For the first flashing stage (C.V.2):

$${ṁ}_{F}={ṁ}_{B_{\left(i\right)}}+{ṁ}_{D_{\left(i\right)}}$$ 3

Balance of dissolved solids for any flashing stage (C.V.3):

$${ṁ}_{B_{\left(i-1\right)}}\times X_{B_{\left(i-1\right)}}={ṁ}_{B_{\left(i\right)}}\times X_{B_{\left(i\right)}}$$ 4

Balance of dissolved solids for the first flashing stage (C.V.2):

$${ṁ}_{F}\times X_{F}={ṁ}_{B_{\left(i\right)}}\times X_{B_{\left(i\right)}}$$ 5

The following eqs 6–12 describe the energy balance in the proposed system. Note that in every heat exchange operation, 10% of the energy is lost to the environment due to the 90% efficiency of the heat exchangers (η_*TC*_).

For the heater (C.V. 1):

$${ṁ}_{S}\times h\left(T_{s}\right)\times \eta_{TC}={ṁ}_{F}(h\times (T_{F_{0}})-h\times (T_{F_{\left(n\right)}}))={\mathrm{Q̇}}_{S}$$ 6

For the first flashing stage (C.V. 2):

$${ṁ}_{F}\times h(T_{F_{0}})={ṁ}_{D_{\left(i\right)}}\times h(T_{D_{\left(i\right)}})+{ṁ}_{B_{\left(i\right)}}h(T_{B_{\left(i\right)}})$$ 7

$${ṁ}_{F}\cdot (h(T_{F_{\left(n\right)}})-h(T_{F_{\left(n-1\right)}}))={ṁ}_{D_{\left(i\right)}}\cdot h_{\mathrm{vap}}(T_{D_{\left(i\right)}})\cdot \eta_{TC}$$ 8

For any flashing stage (C.V. 3):

$${ṁ}_{B_{\left(i-1\right)}}\times h(T_{B_{\left(i\right)}})={ṁ}_{D_{\left(i\right)}}\times h(T_{D_{\left(i\right)}})+{ṁ}_{B_{\left(i\right)}}\times h(T_{B_{\left(i\right)}})$$ 9

$${ṁ}_{F}\cdot (h(T_{F_{\left(n-i\right)}})-h(T_{F_{\left(n-i-1\right)}}))={ṁ}_{D_{\left(i\right)}}\cdot h_{\mathrm{vap}}(T_{D_{\left(i\right)}})\cdot \eta_{TC}+\left(h(T_{D_{\left(i-1\right)}})\cdot \sum_{i=1}^{i-1}{\dot{m}}_{D_{\left(i\right)}}-h(T_{D_{\left(i\right)}})\cdot \sum_{i=1}^{i-1}{ṁ}_{D_{\left(i\right)}}\right)\cdot \eta_{TC}$$ 10

For the last flashing stage (C.V. 4):

$${ṁ}_{B_{\left(n-1\right)}}\times h(T_{B_{\left(n-1\right)}})={ṁ}_{D_{\left(n\right)}}\times h(T_{D_{\left(n\right)}})+{ṁ}_{B_{\left(n\right)}}\times h(T_{B_{\left(n\right)}})$$ 11

$${ṁ}_{F}\cdot (h(T_{F_{\left(1\right)}})-h(T_{CW}))={ṁ}_{D_{\left(n\right)}}\cdot h_{\mathrm{vap}}(T_{D_{\left(n\right)}})\cdot \eta_{TC}+\left(h(T_{D_{\left(n-1\right)}})\cdot \sum_{i=1}^{n-1}{ṁ}_{D_{\left(i\right)}}-h(T_{D_{\left(n\right)}})\cdot \sum_{i=1}^{n-1}{ṁ}_{D_{\left(i\right)}}\right)\cdot \eta_{TC}$$ 12

The equations regarding the mass and energy balance were used in a simplified form to better suit the model assumptions. Their full form and the simplification process can be found in Smith et al.^11^

Equations 13, 14 and 15 will also be applied to solve the mass and energy balance of the proposed system. In this study, a terminal temperature difference of the heater equal to 10 °C (Tayyeban et al.^6^) will be adopted. Furthermore, the sum of losses associated with the demister and the nonequilibrium allowance will be 0.2 °C (El-Dessouky and Ettouney^1^).

$${\mathrm{TTD}}_{h}=T_{S}-T_{F0}$$ 13

$${\mathrm{TTD}}_{c_{\left(1\right)}}=T_{F_{0}}-T_{F_{\left(n\right)}}-\Delta T_{st}-0.213-{\mathrm{EBP}}_{\left(i\right)}$$ 14

$$T_{B_{\left(i\right)}}-T_{D_{\left(i\right)}}=\mathrm{EBP}+0.213$$ 15

To calculate the elevation of the boiling point (EBP) of vinasse, the correlation presented in eq 16 (Starzak et al.,^12^), which estimates the EBP for a sucrose-water solution in °C, will be used.

$${\mathrm{EBP}}_{\mathrm{vin}}=\left[\frac{\left(1+\frac{2121.4}{3797.06}\cdot y^{2}\cdot (1-1.0038\cdot y-0,24653\cdot y^{2})\cdot \frac{T+226.28}{T+273.15}\right)}{1+\frac{T+226.28}{3797.06}\cdot \ln(1-y)}-1\right]\cdot (T+226.28)$$ 16

According to Sanaye and Asgari,^13^ for the modeling of MSF systems, the temperature drop between flashing stages should be equal, especially when aiming to optimize the process. Therefore, eq 17 will also be utilized to solve the mass and energy balance of the proposed system.

$$\Delta T_{st}=T_{B_{\left(i-1\right)}}-T_{B_{\left(i\right)}}$$ 17

### Optimization Algorithm

For the optimization of the variables undefined by the model: number of flashing stages and the terminal temperature drop in the condenser of the first stage (TTD_*c*_(1)__); The proposed model will consider the following objectives:

- Maximization of exergy efficiency
- Maximization of thermal performance ratio
- Minimization of total heat exchange area

Exergy efficiency is broadly used to analyze industrial processes from an environmental and efficiency standpoint. Camargos et al.,^14^ for example, analyzed an annular shaft limekiln working with producer gas as renewable biofuel. Their exergy diagnostic proved important to find optimization opportunities in the proposed process. For the calculation of exergy efficiency (η_*e*_), eq 18 was used. The initial concentration of total dissolved solids in the inlet stream (*X*_*F*_) and the ambient pressure and temperature conditions (25 °C and 1 atm) were taken as the reference state for exergy calculations (absolute dead state). To this end, the exergy balance (eq 19) was applied to all flashing stages (V.C 2, 3, and 4) as well as to the heater (V.C 1). Additionally, the work required for the operation of the pump responsible for feeding the system was considered (eq 20—Amiri Rad et al.^10^).

$$\eta_{e}=1-\left(\frac{İ}{{ė}^{\prime }}\right)$$ 18

$$\sum_{e=1}^{p}{ṁ}_{e}{\left[e_{Q}+e_{\mathrm{Fe}}+e_{M}\right]}_{e}-\sum_{s=1}^{q}{ṁ}_{e}{\left[e_{Q}+e_{\mathrm{Fe}}+e_{M}\right]}_{s}+Ẇ+\sum_{i=1}^{N}{\mathrm{Q̇}}_{i}\cdot \left[1-\frac{T_{0}}{{T_{f}}_{i}}\right]=İ$$ 19

$${Ẇ}_{P}=\frac{{ṁ}_{F}{\mathrm{vol}}_{\mathrm{vin}}(\Delta P)}{\eta_{P}}$$ 20

The specific volume of vinasse (vol_vin_) was assumed to be constant and equal to that of water at 25 °C: 0.0001 m^3^/kg. Since vinasse is predominantly composed of water (Spinelli et al.^8^) and the system does not operate at very high temperatures, this assumption is reasonable. To calculate the pressure increase (Δ*P*), the pressure of the inlet stream was considered equal to atmospheric pressure, while the pumping pressure was set to the saturation state of pure water at the temperature of the inlet stream after heating plus the pressure drop that was considered to be 1 atm. The isentropic efficiency of the pump (η_*P*_), as considered to be 70% (Amiri Rad and Mohammadi^10^).

The flow exergy will be considered in three components: mixing, physical, and chemical. For this, eqs 21, 22 and 23 were used:

$$e_{Q}=\sum_{i=1}^{z}x_{i}\cdot 10^{3}\cdot \frac{e_{Q_{i}}^{{}^{\circ}}}{M_{i}}+RT_{0}\sum_{i=1}^{z}\frac{x_{i}}{M_{i}}\ln(y_{i})$$ 21

$$e_{Fe}=\sum_{i=1}^{z}x_{i}[(h_{i}-h_{0})+T_{0}\cdot (s_{i}-s_{0}){]}_{i}$$ 22

$$e_{M}=h_{\mathrm{Sal}}-\sum_{i=1}^{z}x_{i}\cdot h_{i}-T_{0}\cdot \left(s_{\mathrm{Sal}}-\sum_{i=1}^{z}x_{i}\cdot s_{i}\right)$$ 23

To calculate the chemical component of the flow exergy, the standard chemical exergy of the sucrose (6007.8 kJ/mol—Dogbe et al.^15^) and water (0.9 kJ/mol—Al Ghamdi and Mustafa^16^) were used.

For the mixture component of the flow exergy, the entropy (392.40 J/(molK) at 25 °C and 1 atm—Lambert and Leff^17^) and enthalpy (183.15 kJ/kg at 25 °C and 1 atm—Lambert and Leff^17^) of the solid sucrose were used. The molar mass value of sucrose required to convert specific values from a molar basis to a mass basis used was: 342.2965 g/mol (Perry and Green^18^).

Therefore, the total destroyed exergy (eq 24) and the total input exergy for the system (eq 25) can be calculated.

$${İ}_{T}=\sum_{i=1}^{n}{İ}_{\left(i\right)}+{İ}_{h}+W_{P}$$ 24

$${ė}^{\prime }={\mathrm{Q̇}}_{S}\cdot \left[1-\frac{T_{0}}{T_{S}}\right]+{ṁ}_{F}{\left[e_{Q}+e_{\mathrm{Fe}}+e_{M}\right]}_{F}$$ 25

Equation 25 accounts for the flow exergy of the feed stream and the indirect heat transferred from the condensation of VG3 in the heater.

The thermal performance ratio (eq 26) represents the ratio between the mass flow rate of distillate water and the mass flow rate of steam used for heating the inlet stream (Tayyeban et al.^6^):

$$\mathrm{PR}=\frac{\sum_{i=1}^{n}{ṁ}_{D_{\left(i\right)}}}{{ṁ}_{S}}$$ 26

The calculation of the total heat exchange area includes the sum of the condenser areas for each flashing stage and the heater area (eq 27).

$$A_{T}=A_{h}+\sum_{i=1}^{n}A_{c_{\left(i\right)}}$$ 27

The heat exchange areas can be calculated using eqs 28 (condenser) and 29 (heater) (El-Dessouky and Ettouney^1^):

$$A_{c_{\left(i\right)}}=\frac{{ṁ}_{D_{\left(i\right)}}h_{\mathrm{vap}}(T_{D_{\left(i\right)}})}{U_{c_{\left(i\right)}}\times {\mathrm{LMTD}}_{c_{\left(i\right)}}}$$ 28

$$A_{h}=\frac{{ṁ}_{S}h_{\mathrm{vap}}(T_{S})}{U_{h}\times {\mathrm{LMTD}}_{h}}$$ 29

Equation 30 was used to estimate the overall heat transfer coefficient (El-Dessouky and Ettouney^1^):

$$U=10^{-3}\left(1719.4+3.2063T+0.015971T^{2}-0.00019918T^{3}\right)$$ 30

The validation was performed by analyzing the percentage deviation between the parameters calculated using the proposed model and the models proposed by Helal^19^ and El-Dessouky and Ettouney.^1^ Since their models corresponds to systems operating with seawater, the Equations in this model that relate to the physicochemical parameters of vinasse, were respectively replaced to describe the behavior of seawater.

Equations 31 (specific inlet flow rate) and 32 (specific heat exchange area), that are used to evaluate the performance of MSF system, were used in the validation of this model.

$$s{ṁ}_{F}=\frac{{ṁ}_{F}}{\sum_{i=1}^{n}{ṁ}_{D_{\left(i\right)}}}$$ 31

$$ssA=\frac{A_{T}}{\sum_{i=1}^{n}{ṁ}_{D_{\left(i\right)}}}$$ 32

For the optimization, the *gamultiobj* function in MATLAB software was used. It utilizes a version of the NSGA-II algorithm, which is classified as controlled and elitist. Thus, in addition to always selecting individuals with the highest rank (elitist), it also prioritizes the selection of solutions that can enhance population diversity, even if these solutions have a low ranking. These algorithms are well-known in the multiobjective optimization of industrial process. Leite et al.^20^ applied Generalized Differential Evolution 3, another type of genetic algorithm, to optimize an adiabatic styrene reactor. For this work, the limits selected for the two variables of interest were:

- *n*: [6;60]
- TTD_*c*_(1)__: [3;15]

Although according to El-Dessouky and Ettouney,^1^ the terminal temperature difference of the condenser typically falls within the range of 3 to 5 °C in MSF plants, literature presents examples that contradict this claim (Helal^19^). Therefore, this parameter was investigated over a broader range in this study. Equation 33 represents the algebraic form of the optimization function proposed in this study. It is noted that the objectives intended for maximization are accompanied by a negative sign due to the inherent nature of the gamultiobj function, which by default minimizes all objectives.

$$\mathrm{gamultiobj}(-\mathrm{PR},-\eta_{e},A_{T})=\mathrm{Pareto}\;\mathrm{front}$$ 33

To select the best option among the optimal solutions on the Pareto front, the TOPSIS decision-making algorithm was used (Tayyeban et al.^6^). The weights used by the algorithm are

- Exergy efficiency: 0.3
- Thermal performance ratio: 0.4
- Total heat exchange area: 0.3

The selection of these weight values was based on the premise that the main objective of the system is to concentrate the sugar cane vinasse; therefore, the optimal solution should prioritize the recovery of water.

## Results and Discussion

Table 2 presents the comparison between the results of the proposed model and those of the MSF-OT present in the literature (eq 34). It can be observed that there are slight deviations in the parameters compared, with the most significant one being the heat exchange area.

$$\mathrm{Deviation}\;(\%)=\left(\frac{\mathrm{Proposed}\;\mathrm{model}}{\mathrm{Literature}\;\mathrm{model}}\right)-1$$ 34

**Table 2:** Comparison between the Proposed Model and the MSF-OT Plant Models Present in the Literature

parameters	El-Dessouky and Ettouney^1^	proposed model	deviation (%)	Helal^19^	proposed model	deviation (%)
*PR*	3.96	4.09	3.28%	10.00	10.20	2.00%
*sA*	117.15	112.59	–3.89%	212.90	294.27	38.22%
*sṁ*_*F*_	8.93	9.27	3.81%	8.05	7.93	–1.49%
*ṁ*_*S*_	95.49	89.21	–6.58%	87.60	87.26	–0.39%
*A*_*T*_	44,378	41,099	–7.39%	186,504	261,760	40.35%
$\sum_{i=1}^{n}{\dot{m}}_{D_{\left(i\right)}}$	378.80	365.05	–3.63%	876.02	889.51	1.54%

The small variations observed in relation to the El-Dessouky and Ettouney^1^ model were anticipated, since their model assumes that the specific heat capacity at constant pressure of liquid streams remains constant and equals 4.18 kJ/(kg °C). In contrast, the model proposed in this study uses correlations to estimate the *C*_*P*_ of seawater and condensed water streams, considering their temperatures and, in the case of seawater, the salt concentration.

In relation to the Helal^19^ model, the significant variation observed in the thermal exchange area parameter can be explained by the fact that the model employs a more robust formulation for calculating the global heat transfer coefficient. This model accounts for different thermal resistances that influence this parameter, such as those associated with fouling and the film coefficients of seawater and condensate. However, this level of precision is not pertinent to the present study.

The Pareto front resulting from the optimization routine, as well as the optimal point determined by the TOPSIS decision-making algorithm, are presented in Figure 2. It was determined that the optimal number of flashing stages is 58, and the terminal temperature difference of the condenser in the first stage is 4.3 °C. These parameters optimize the total heat exchange area, exergy efficiency, and thermal performance ratio of the proposed plant.

**Figure 2:**
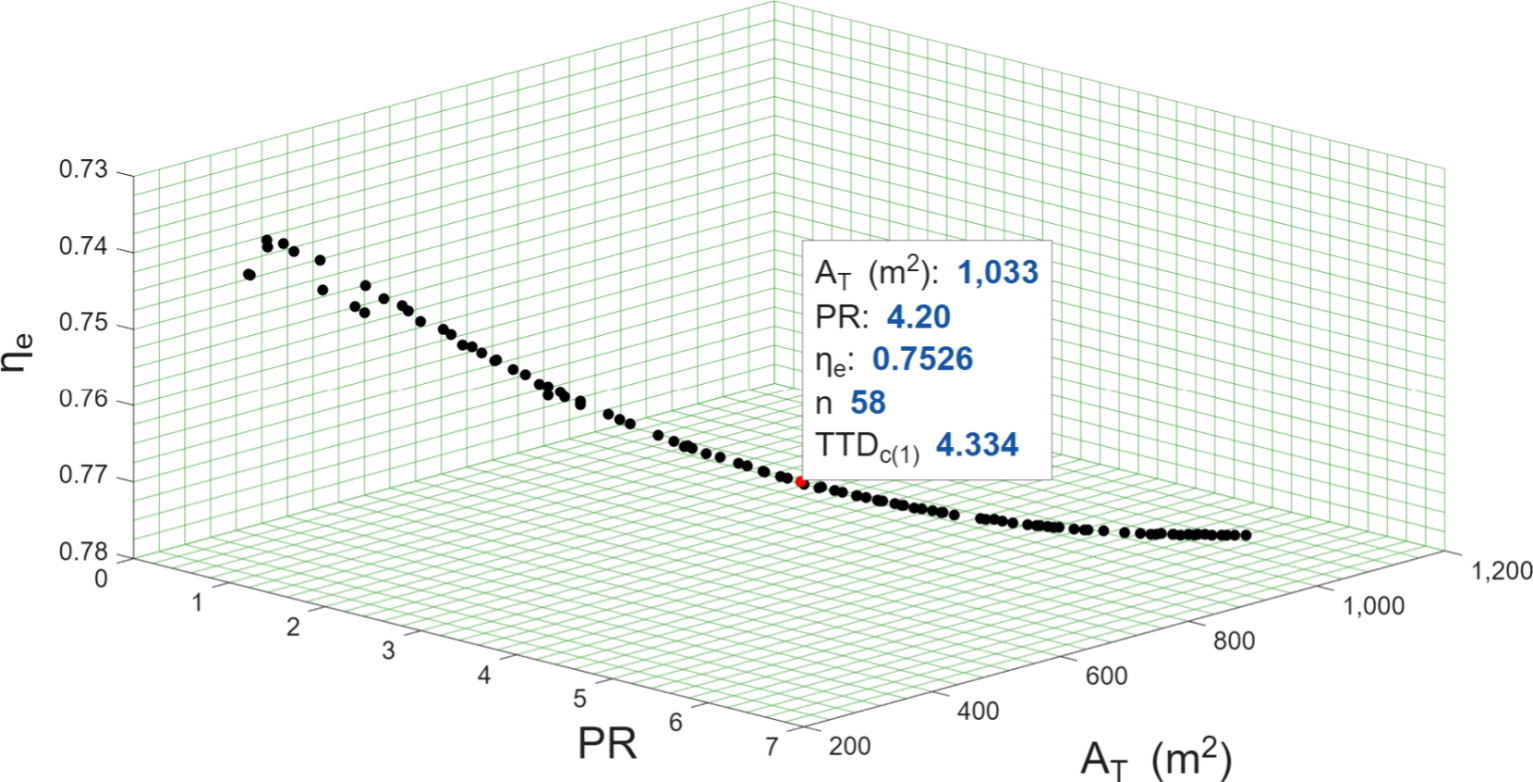
Pareto front and optimal point according to the TOPSIS decision-making algorithm.

Table 3 presents the values of some parameters of interest for the analysis of the optimized proposed model.

**Table 3:** Parameters of the Optimized Proposed Model

parameters	results
*A*_*T*_ [m^2^]	1033
η_*e*_ [%]	0.7526
*PR* [*−*]	4.202
*sṁ*_*F*_ [*−*]	23.53
*ṁ*_*S*_ [kg/s]	0.6270
*sA* [m^2^/(kg/s)]	392.0
$\sum_{i=1}^{n}{\dot{m}}_{D_{\left(i\right)}}\;[\mathrm{kg}/s]$	2.635
*X*_*B*_(*n*)__ [PPM]	39,828
*T*_*B*_(*n*)__ [°C]	63.6
*ṁ*_B_(n)__ [kg/s]	59.37

Regarding the total heat exchange area, the obtained value is significantly lower than those reported by El-Dessouky and Ettouney^1^ and Helal.^19^ This can be attributed to the feed flow rate of vinasse, which is approximately 55 times lower than in the plant model of El-Dessouky and Ettouney^1^ and about 114 times lower than in the Helal^19^ model. With a lower feed flow entering the system, there is reduced steam demand for heating and less flash generation in the stages, thereby decreasing the required heat exchange area. In the model proposed by Tayyeban et al.,^6^ the determined heat exchange area was 1133 m^2^, a result consistent with the findings of this study.

The exergy efficiency obtained is superior to that found in the model by Tayyeban et al.,^6^ which reported a value of 61.58%. The inlet flow of vinasse into the system at a temperature higher than ambient significantly contributes to the increase in exergy efficiency, as it minimizes heat exchange operations and consequently reduces the irreversibilities associated with these processes.

Regarding the thermal performance ratio, the obtained result is like that presented in the MSF-OT plant model developed by El-Dessouky and Ettouney,^1^ making it a reasonable outcome. However, it indicates a low conversion of heating steam into distilled water, which may discourage the application of the proposed system. In the model proposed by Tayyeban et al.,^6^ also designed for the treatment of industrial wastewater streams, the PR obtained was 6.21.

The optimized system distilled water production rate is 228 m^3^/day or 50,080 m^3^/year, assuming a 220-day harvest period. When compared to the system described by Cruz et al.,^9^ the optimal system produces 85% less distilled water. However, the low °Brix of the vinasse at the outlet reduces the risk of scaling or spontaneous crystallization within the equipment.

The number of flashing stages found in the proposed system is considered high for an MSF-OT project, but this is counterbalanced by the total heat exchange area, which is significantly lower than usual. Comparing it to the system proposed by Helal,^19^ which includes 25 flashing stages, the optimized system has only 0.6% of the heat exchange area of the model proposed by Helal,^19^ despite featuring 33 additional flashing stages. Furthermore, the low vinasse flow rate and high input temperature require more stages to ensure that all flashed vapor is condensed. It is theorized that, if the selected MSF system includes heat rejection stages, that operate with cold water to condensate the distilled water, the total number of required flashing stages would be lower.

Figures 3 and 4 illustrate, respectively, the influence of the number of flashing stages and the terminal temperature difference in the condenser of the first stage on the total heat exchange area, exergy efficiency, and thermal performance ratio of the proposed plant.

**Figure 3:**
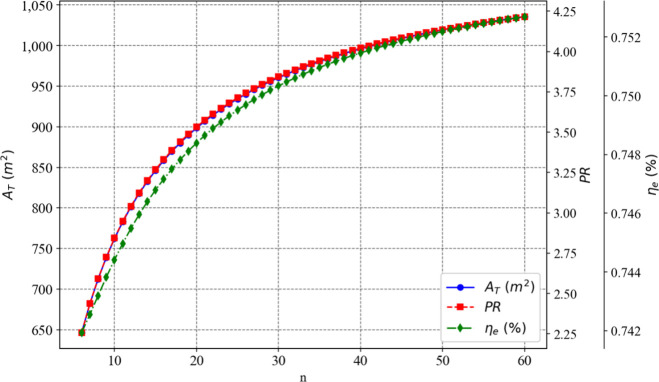
Effect of the number of flashing stages on the optimization objectives while TTD_*c*_(1)__ remains constant at 4.3 °C.

**Figure 4:**
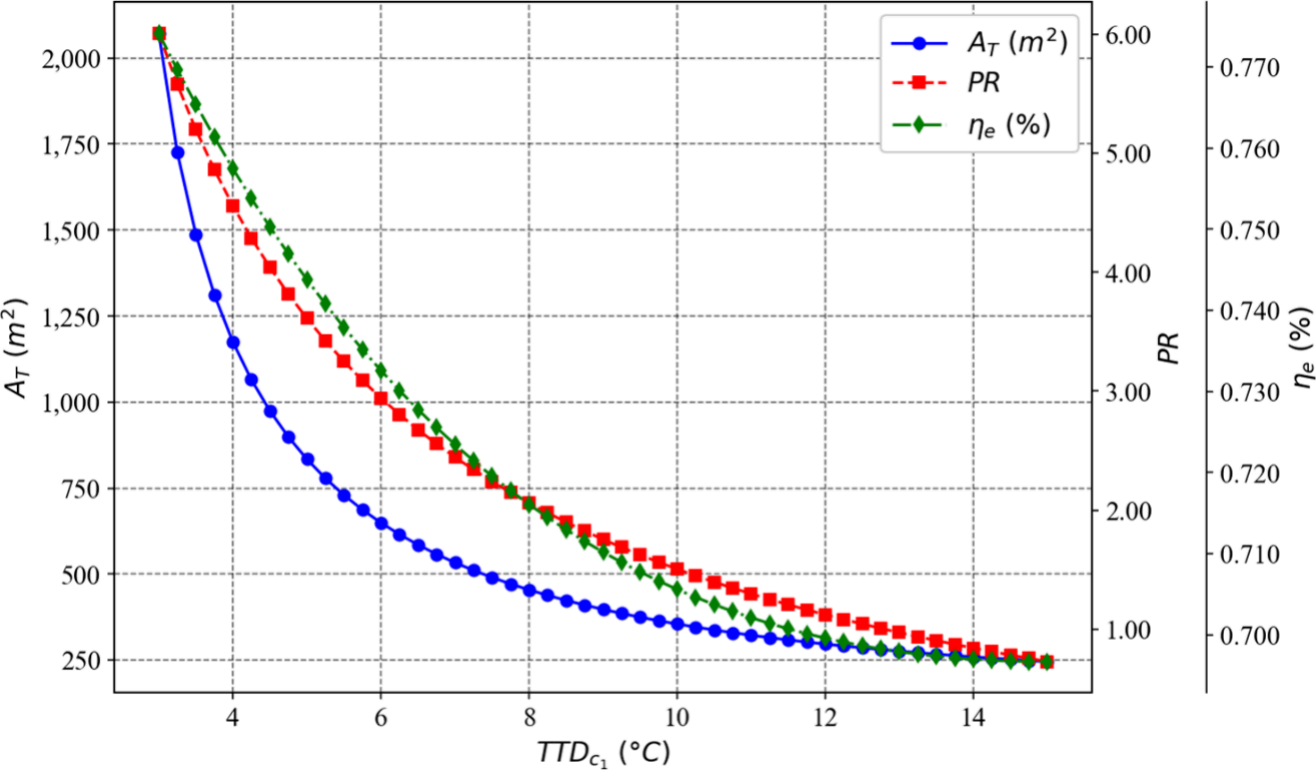
Effect of TTD_*c*_(1)__ on the optimization objectives while the number of flashing stages remains fixed at 58.

It can be observed that as the number of stages increases, the values of the three objectives also rise. Conversely, the higher the terminal temperature difference of the condenser in the first stage, the lower the values of the three objectives. Thus, the choice to minimize the total heat exchange area as one of the objectives proved to be appropriate, as it moves in the opposite direction to the other two objectives.

Regarding the total distillate flow rate, the value is considerably small, even when compared to systems with an equivalent total heat exchange area, such as the system by Tayyeban et al.,^6^ which generates 35 kg/s of distilled water. This discrepancy occurs due to the lower vinasse input flow rate relative to those other systems, as well as the choice of the MSF-OT process, which does not include heat rejection stages to treat the vinasse. The vinasse enters the system at a considerably high temperature, resulting in low distilled water production. The higher the temperature of the preheated input stream in any stage, the lower the flash flow rate that the stage can generate, as complete condensation of this vapor is one of the premises of this mathematical model.

Figure 5 presents the variation of the objectives in relation to the inlet stream temperature for the optimized system.

**Figure 5:**
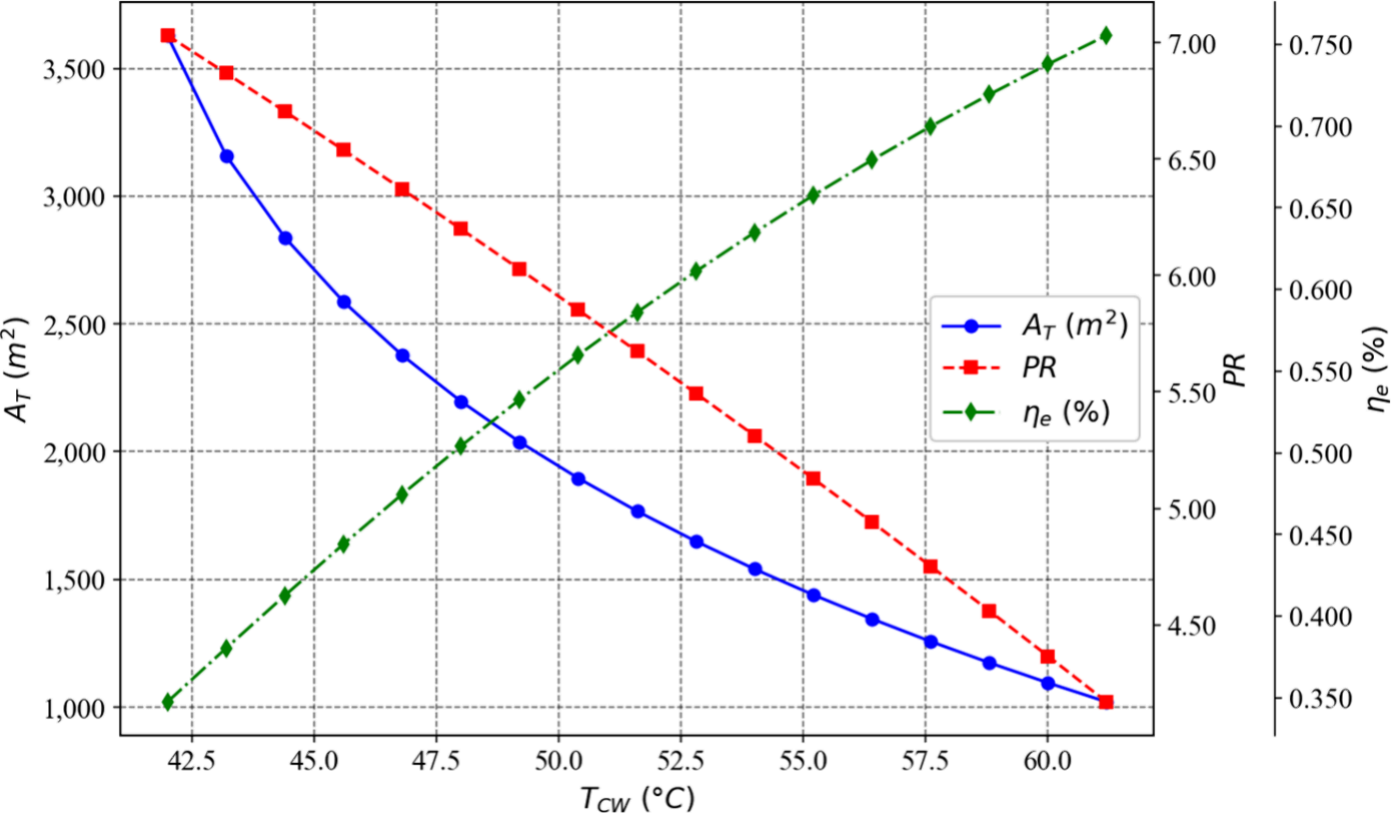
Effect of input stream temperature on optimization objectives.

It is notable that a lower input stream temperature decreases the thermal performance ratio, supporting the thesis that, if the system included heat rejection stages, the production of distilled water would be higher. The opposite effect is observed in exergy efficiency, whose decline can be attributed to the increase in heat exchange operations, generating more irreversibilities. The heat exchange area increase is associated with a larger heater area due to the lower temperature of the preheated input stream, which also contributes to the increased areas of the condensers.

Therefore, the plant features relatively low capital costs, making it a viable opportunity for sugar cane processing facilities seeking to meet ESG objectives or facing challenges in obtaining water.

## Conclusions

Through the optimization routine, it was determined that, for the plant that provided data for this study, the optimal number of flashing stages and the terminal temperature difference of the condenser in the first stage that maximize exergy efficiency and thermal performance ratio while minimizing the total heat exchange area are 58 and 4.3 °C, respectively. Under these conditions, the three key performance indicators are 75.26%, 4.202, and 1033 m^2^, respectively.

The high number of stages is balanced by the relatively small total heat exchange area, resulting in an expected lower CAPEX design compared to MSF-OT plants with fewer flashing stages. Therefore, it is expected that the implementation of the optimal system in feasible in the optics of the required CAPEX.

Under ideal conditions, the model presents an efficient total heat exchange area, reducing the capital costs of the project. The high exergy efficiency indicates minimal irreversibilities within the proposed system. However, the relatively low thermal performance ratio is due to the high temperature of the inlet stream and the absence of heat rejection stages in the model. The optimized system would be capable of producing 228 m^3^/day or 50,080 m^3^/year of distilled water, assuming a 220-day harvest season. Although this output is lower than that of current market concentration systems, the low final °Brix of the vinasse minimizes the risk of scaling or spontaneous crystallization within the equipment, reducing costs with maintenance. Given the relatively small total heat exchange area, the system presents a compelling opportunity for recovering water for sugar cane processing in regions where this resource is scarce and to advance ESG initiatives.

## Nomenclature

*A*: heat exchange area (m^2^)
*Q̇*: heat (kJ/s)
*U*: overall heat transfer coefficient ( $\frac{\mathrm{kW}}{{\mathrm{Km}}^{2}}$ )
*LMTD*: logarithmic mean temperature difference (°C)
*sṁ*_*F*_: specific flow rate (−)
*sA*: specific heat exchange area $\left(m^{2}/\frac{\mathrm{kg}}{s}\right)$
*TTD*_*C*_: terminal temperature difference in the condenser (°*C*)
η_*e*_: exergetic efficiency (%)
η_*TC*_: heat exchanger efficiency (%)
η_*P*_: isentropic pump efficiency (%)
*y*: mole fraction of sucrose (−)
*x*: mass fraction of sucrose (−)
*EBP*: elevation of the boiling point (kJ/kg)
*h*: specific enthalpy (kJ/kg)
*h*_*vap*_: specific enthalpy of vaporization (kJ/kg)
*e*_Fe_: flow exergy—physics (kJ/kg)
*e*_*M*_: flow exergy—mixture (kJ/kg)
*e*_*Q*_: flow exergy—chemistry (kJ/mol)
*ė*′: total exergy entering the system (kJ/s)
VG3: low temperature steam in saturation state from the concentration of sugar cane juice (−)
*PR*: thermal performance ratio (−)
*X*_*F*_: total soluble solids in the vinasse inlet stream (%)
*X*_*B*_: total soluble solids in the concentrate vinasse stream (%)
*İ*: exergy destruction rate (kJ/s)
*T*_*fi*_: border temperature (°C)
*T*_*F*_0__: top brine temperature (°C)
*T*_*CW*_: vinasse inlet temperature (°C)
*T*_*D*_: distilled water temperature (°C)
*T*_*F*_: cold vinasse temperature (°C)
*T*_*B*_: concentrated vinasse temperature (°C)
*vol*_*vin*_: specific volume of vinasse (m^3^/kg)
ΔP: pressure difference (kPa)
*T*_0_: dead state temperature (°C)
*Ẇ*: work (kJ/s)
ΔT_*st*_: temperature drop per flashing stage (°C)
*ṁ*_*D*_: mass flow rate of distilled water (kg/s)
*ṁ*_*F*_: mass flow rate of vinasse (kg/s)
*ṁ*_*B*_: mass flow rate of concentrated vinasse (kg/s)
*ṁ*_*S*_: mass flow rate of VG3 (kg/s)
